# In-Season Estimation of Japanese Squash Using High-Spatial-Resolution Time-Series Satellite Imagery

**DOI:** 10.3390/s25071999

**Published:** 2025-03-22

**Authors:** Nan Li, Todd H. Skaggs, Elia Scudiero

**Affiliations:** 1 Environmental Sciences, University of California, Riverside, CA 92521, USA; 2 USDA-ARS U.S. Salinity Laboratory, Riverside, CA 92507, USA; todd.skaggs@usda.gov

**Keywords:** remote sensing, yield estimation, satellite imagery, NDVI

## Abstract

Yield maps and in-season forecasts help optimize agricultural practices. The traditional approaches to predicting yield during the growing season often rely on ground-based observations, which are time-consuming and labor-intensive. Remote sensing offers a promising alternative by providing frequent and spatially extensive information on crop development. In this study, we evaluated the feasibility of high-resolution satellite imagery for the early yield prediction of an under-investigated crop, Japanese squash (*Cucurbita maxima*), in a small farm in Hollister, California, over the growing seasons of 2022 and 2023 using vegetation indices, including the Normalized Difference Vegetation Index (NDVI) and the Soil-Adjusted Vegetation Index (SAVI). We identified the optimal time for yield prediction and compared the performances across satellite platforms (Sentinel-2: 10 m; PlanetScope: 3 m; SkySat: 0.5 m). Pearson’s correlation coefficient (*r*) was employed to determine the dependencies between the yield and vegetation indices measured at various stages throughout the squash growing season. The results showed that SkySat-derived vegetation indices outperformed those of Sentinel-2 and PlanetScope in explaining the squash yields (R^2^ = 0.75–0.76; RMSE = 0.8–1.9 tons/ha). Remote sensing showed very strong correlations with yield as early as 29 days after planting in 2022 and 37 and 76 days in 2023 for the NDVI and the SAVI, respectively. These early dates corresponded with the vegetative stages when the crop canopy became denser before fruit development. These findings highlight the utility of high-resolution imagery for in-season yield estimation and within-field variability detection. Detecting yield variability early enables timely management interventions to optimize crop productivity and resource efficiency, a critical advantage for small-scale farms, where marginal yield changes impact economic outcomes.

## 1. Introduction

Pre-harvest crop yield predictions, particularly at the field level, may help farmers optimize in-season agronomic management, possibly increasing agricultural productivity and sustainability. These strategies include understanding the outcomes of different planting methodologies, irrigation scheduling [1], the judicious application of fertilizers [2], and pest management [3]. These management decisions directly affect farming profitability and its environmental footprint [4,5]. Moreover, accurate in-season yield estimations may provide farmers and the agricultural industry with the information used for risk assessment, determining insurance premiums, and evaluating input costs. Stakeholders along the agricultural chain can make informed decisions using timely and comprehensive data on crop yield variability [6]. For policymakers, early yield predictions offer insights into food security and resilience against potential market fluctuations [7]. Notably, the earlier in the growing season yield information is accurately forecasted, the more effective agriculture practices are likely to be, underscoring the importance of continued investment in research and technology development in this critical area of agricultural science [7]. Traditionally, to predict the crop yield, field surveys and crop cuttings are carried out near the harvest period to estimate the final yield. However, these approaches are somewhat time-consuming and resource-intensive regarding labor and financial investment and only provide a limited window for decision-making.

Remote sensing methods, which use multi-spectral satellite images, facilitate the characterization of crop yield patterns over space and time and expand the scope of crop monitoring beyond traditional field observations [8]. The visible red, green, and blue bands and near-infrared (NIR) spectral bands provide information on vegetation conditions. Vegetation indices such as the Normalized Difference Vegetation Index (NDVI) combine reflectance from multiple bands and are particularly useful for yield estimation because they integrate key biophysical and biochemical characteristics. Among the many available vegetation indices, the NDVI and the Soil-Adjusted Vegetation Index (SAVI) were chosen for this study. The NDVI is widely used in yield prediction due to its ease of use, reliability, and strong correlation with crop biomass and production [9,10]. Because it accurately reflects canopy density and photosynthetic activity, the NDVI works particularly effectively in farmland with dense vegetation. On the other hand, the SAVI minimizes the soil background effects, making it suitable for early growth stages and sparse canopies [11]. Indices such as the Enhanced Vegetation Index (EVI), the Green Chlorophyll Vegetation Index (GCVI), and Near-Infrared Reflectance of Vegetation (NIRv) also have applications in yield estimation, but they were not selected for this study. The EVI is useful in high-biomass scenarios, but it requires additional atmospheric correction and is less effective in the early growth stages. While the GCVI and NIRv are sensitive to chlorophyll content and photosynthesis, they are less commonly used in small-scale agriculture systems. In this study, we selected the NDVI and the SAVI because of their robustness, simplicity, and proven effectiveness in agricultural applications. 

By integrating remote sensing data with environmental and management variables, recent advances in data-driven methodologies, including machine learning and regression models, have further enhanced the accuracy of yield predictions [12]. The effectiveness of remote sensing for yield prediction is highly dependent on the spatial and temporal resolution of imagery. Although large-scale crop growth monitoring has prompted the extensive use of remote sensing platforms like Sentinel-2 (10 m) and Landsat (30 m), their spatial resolution is insufficient to capture within-field short-scale variability, especially in small-scale farming systems [13,14]. The new generation of nanosatellites, such as the PlanetScope CubeSat constellation, provide cost-effective, near-nadir-view coverage of the Earth with a high spatial resolution of around 3 m [15]. Similarly, SkySat, operating in the same sun-synchronous orbit at an altitude of approximately 500 km, captures images with sub-meter spatial resolution. These high-resolution systems are especially valuable for small-scale farm yield predictions, as they detect the fine-scale variations in crop health that are often indicative of yield potential [16,17].

This study aimed to evaluate remote sensing-based yield prediction in small farms. Very high-resolution remote sensing offers frequent crop growth and heterogeneity monitoring. However, there is limited research on the comparative performances of different resolutions for in-season yield prediction in small farms with heterogeneous crop cover (e.g., small plots planted with the same crop). This study offers insights into using Sentinel-2, PlanetScope, and SkySat for the yield prediction of Japanese squash in a small farm in California. Specifically, the objectives of this study were to (i) assess the ability of the NDVI and the SAVI to capture growth dynamics and yield variability at different spatial scales, (ii) identify the optimal timing for accurate yield forecasting, and (iii) compare the in-season yield estimates from publicly available Sentinel-2 imagery with the estimates from high-resolution PlanetScope and SkySat imagery. The findings of this study will provide novel information into the utility of high-resolution, frequently captured spaceborne imagery for small-scale yield prediction. This research offers actionable guidance on crop monitoring for farmers, consultants, and other stakeholders.

## 2. Materials and Methods

### 2.1. Study Site

**Figure 1:**
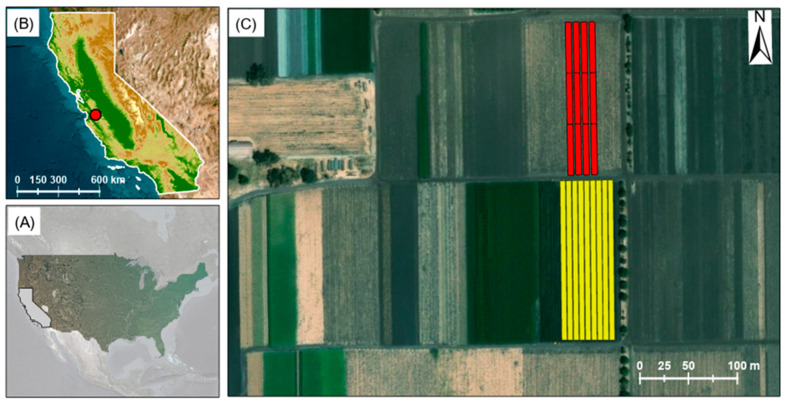
The base maps of (**A**) the United States and (**B**) California. (**C**) A blow-up of the study site in central California. The experimental fields are demarcated in red (2022) and yellow (2023).

This study was conducted on the Phil Foster Ranches (https://www.pinnacleorganic.com/, accessed on 17 January 2025) located ~10 km east of Hollister, California (Figure 1). Soils in the farm are fertile, with generally medium-to-heavy texture, made mostly of silty clay loam and clay, with moderate-to-good drainage (Web Soil Survey, https://websoilsurvey.sc.egov.usda.gov/, accessed on 17 January 2025). The climate in the study area is Mediterranean, characterized by warm and dry summers and mild winters. In summer, the average temperature is around 22 °C, while in winter, it reaches an average of around 5 °C. Rainfall is limited, with an annual average of 380 mm. The area benefits from year-round sunshine, with precipitation occurring on approximately 56 days every year.

### 2.2. Field Experiment Design

The field experiments were carried out in the growth seasons of 2022 and 2023, with the experimental strips oriented north–south. In 2022, the experimental field comprised 12 distinct strips, each measuring 5 m in width and 50 m in length. The strips were delineated to ensure uniformity and facilitate accurate comparisons between the reduced-tillage and no-till treatments. A Falc spader, which operates with ten 15-cm-wide spades mounted on a linkage attached to a horizontal crankshaft, was used to complete the full tillage treatment. This mechanism works at a depth of 30 cm across the entire 2 m bed by driving the spades in and out of the ground in a back-and-forth motion. On the other hand, the reduced tillage treatment used an Orthman strip till to create two 25-cm-wide strips on each 2-m bed to an approximate depth of 3 cm. In order to disturb the soil minimally compared to that of the full tillage approach, each 25-cm strip also had a central chisel shank that penetrated to a depth from 10–15 cm. The 2023 experiment consisted of 9 distinct strips, each measuring 5-m wide and 150-m long. The treatments included winter fallow, strip, and spade full tillage methods. The crops were planted on 18 May for the 2022 growing season, while it was 15 May for the 2023 growing season. For each strip, Japanese squash (*Cucurbita maxima*), a sweet variety in the Cucurbitaceae family, was planted in two rows. The dates of harvesting were 16 August 2022, and 28 August 2023. The squash was harvested by hand, and fresh weight was measured on-site.

### 2.3. Satellite Image Acquisition and Processing

#### 2.3.1. Sentinel-2

Sentinel-2 was launched by the European Space Agency (ESA) to systematically acquire optical imagery at a high spatial resolution over land and coastal waters to support the monitoring of Earth’s surface changes. To revisit sites frequently and acquire data easily, two identical Sentinel-2 satellites (Sentinel-2A and Sentinel-2B) operate together. The satellites are phased 180 degrees from each other in the same orbit. This allows for what would be a 10-day revisit cycle to be completed in 5 days. The Sentinel-2 satellites each carry a single multi-spectral instrument (MSI) with 13 spectral channels in the visible/near-infrared (VNIR) and short-wave infrared spectral ranges (SWIR). Within the 13 bands, the visible and NIR bands are sampled at 10 m, the red edge and SWIR bands are sampled at 20 m, and the atmospheric bands are sampled at 60 m spatial resolution. This comprehensive spectral information significantly improves the ability to detect spatial and temporal fluctuations, as well as variations in vegetation status [18]. The Sentinel-2 imagery was preprocessed using the Sentinel-2 Toolbox (https://sentinel.esa.int/web/sentinel/toolboxes/sentinel-2, accessed on 10 August 2024) within the Google Earth Engine platform (https://developers.google.com/earth-engine/datasets/catalog/COPERNICUS_S2, accessed on 10 August 2024). The preprocessing steps included cloud masking, atmospheric correction, and resampling to a consistent spatial resolution (10 m). Cloud masking was performed using the Sentinel-2 Quality Assessment Band (QA60) to remove the pixels affected by clouds and shadows. Atmospheric correction was applied using the Sen2Cor processor (https://step.esa.int/main/snap-supported-plugins/sen2cor/, accessed on 10 August 2024), which converts Top-of-Atmosphere reflectance to Bottom-of-Atmosphere reflectance.

#### 2.3.2. PlanetScope

PlanetScope is a CubeSat 3U form factor (10 cm × 10 cm × 30 cm) satellite constellation operated by Planet Labs, Inc. (Planet Labs, San Francisco, CA, United States). The PlanetScope constellation consists of approximately 130 satellites, with the capability to image the entire land surface of the Earth nearly daily (equating to a daily collection capacity of 200 million km^2^/day). The PlanetScope satellites produce daily imagery with 4 spectral bands, blue (455–515 nm), green (500–590 nm), red (590–670 nm), and near-infrared (NIR, 780–860 nm). These have a Ground Sampling Distance of 3.0–4.1 m at the nadir and a positional accuracy of <10 m RMSE (Planet Team 2018). The surface reflectance products that have been atmospherically corrected by Planet Labs using the 6SV2.1 radiative transfer model with the ancillary data from a moderate-resolution imaging spectroradiometer [15] were used for this study. Such high-temporal and high-spatial-resolution data are useful for computing plant biomass and crop biophysical characteristics for precision agriculture purposes.

#### 2.3.3. SkySat

The SkySat constellation (Planet Labs, San Francisco, CA, United States) includes 21 high-resolution earth imaging satellites capable of recording panchromatic and multi-spectral images at spatial resolutions of 0.57–0.86 m and 0.75–1.0 m, respectively, depending on the satellite generation [15]. The panchromatic image is a single-band grayscale image covering the spectral range of 450–900 nm. The multi-spectral image consists of four multi-spectral bands (blue, green, red, and NIR). The fleet of 21 high-resolution SkySat satellites became fully operational in the fall of 2020. for most regions on Earth, at least one cloud-free image is available every 7 to 10 days, which allows for monitoring crop growth across diverse environments over an entire growing season with identical measurement protocols. The satellite data were delivered as a 4-band multi-spectral SkySat Ortho Analytic Surface Reflectance product, already pansharpened to 0.5 m and corrected to Bottom-of-Atmosphere reflectance. The cloud-free SkySat ortho-pansharpened images were ordered and downloaded through the Planet platform using the field’s polygon to determine the region of interest.

In this study, the Sentinel-2 images were downloaded and preprocessed within the Google Earth Engine platform (https://developers.google.com/earth-engine/datasets/catalog/COPERNICUS_S2_SR_HARMONIZED, accessed on 10 August 2024). While the cloud-free PlanetScope multi-spectral surface reflectance images were downloaded using Planet’s API, the SkySat ortho-pansharpened images were ordered and downloaded through the Planet platform. To address mixed-pixel effects in the coarser-resolution satellite data (e.g., Sentinel-2), strip boundaries were georeferenced and aligned with imagery grids using ArcGIS software. Additionally, a 1 m internal buffer was applied during analysis to exclude the edge pixels likely to overlap with adjacent treatments or bare soil. Only those with ≥75% of the area overlapped with the buffered zone were retained for further analysis. The pixels that did not meet this purity threshold or that intersected non-target features were systematically masked and excluded from the correlations analysis between vegetation index and yield. This approach ensured that the derived spectral data primarily reflected the target crop area, minimizing contamination and enhancing the reliability of the analysis. For the 2022 growing season, as shown in Table 1, this resulted in three separate time series for the subsequent study, including (1) Sentinel-2 (19 images, 10 m resolution), (2) PlanetScope (67 images, 3 m resolution), and (3) SkySat (4 images, 0.5 m resolution). For the 2023 growing season, 16 Sentinel-2, 66 PlanetScope, and 15 SkySat images were selected for analysis.

**Figure 2:**
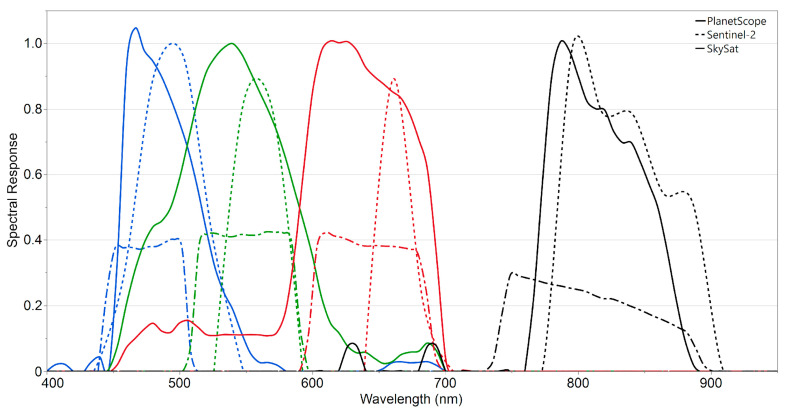
The spectral responses of Sentinel-2 (dotted lines), PlanetScope (solid lines), and SkySat (dot-dash lines) in the blue, green, red, and infrared bands. (Source: https://sentinels.copernicus.eu/web/sentinel/user-guides/sentinel-2-msi/document-library/-/asset_publisher/Wk0TKajiISaR/content/sentinel-2a-spectral-responses and https://support.planet.com/hc/en-us/articles/360014290293-Do-you-provide-Relative-Spectral-Response-Curves-RSRs-for-your-satellites-, accessed on 15 August 2024).

Figure 2 shows that Sentinel-2, PlanetScope, and SkySat have different spectral response functions. Notably, the spectral response functions for the three visible PlanetScope bands spectrally overlap. In addition, the PlanetScope bands are broader with nominal bandwidths of 440–560 nm (blue), 450–650 nm (green), 570–700 nm (red), and 760–890 nm (NIR). The Sentinel-2 bands are narrower, with nominal bandwidths of 440–550 (blue), 525–600 (green), 640–700 (red), and 770–910 (NIR). The SkySat bands are narrower and similar to the PlanetScope; however, the spectral values are less than half of the PlanetScope and Sentinel-2 bands. The crop growth-related vegetation indices were derived from a combination of spectral bands (i.e., Green, Red, and NIR bands) that all three sensors have in common and used to estimate yield. Sentinel-2, PlanetScope, and SkySat have different spectral band wavelengths; however, the NDVI and the SAVI are comparable across these datasets due to the normalization process employed in these indices. The differences in wavelength ranges were accounted for by using the same bands (red and NIR) across all the sensors [19].

### 2.4. Vegetation Indices

The Normalized Difference Vegetation Index (NDVI) was designed to monitor biomass and exploit the difference between the red and NIR bands for heavily vegetated land cover types. It has also been shown to be strongly correlated with plant productivity (Table 2). The NDVI is often preferred over the independent use of the red and NIR channels in that it simplifies data analysis into a single metric, while at the same time, it represents normalization using the reflectance values in multiple wavebands, which is effective in reducing the influence of errors or uncertainty due to atmospheric and background differences, as well as enhancing and/or linearizing the spectral response to target vegetation [5,8]. Normalization also allows for easier comparison across different sensors [4]. High NDVI values refer to green and healthy plants, while low NDVI values indicate that the plant is not green or has low amounts of vegetation. Another vegetation index used in this study was the Soil-Adjusted Vegetation Index (SAVI), which was designed to minimize the effect of soil reflection on red and near-infrared radiation by adding an estimated background correction factor [11]. The SAVI is ideal for the early stages of crop growth and for monitoring crops that do not cover the soil, even in the most advanced growth stages [19].

### 2.5. Analysis and Evaluation

For each date of acquisition, linear regression was performed, and a coefficient of determination (R^2^) was calculated. The best date in terms of maximum Pearson’s correlation coefficient (*r*) was determined to estimate crop yields. Statistical comparisons were carried out between the yield and vegetation indices, and the significance of these relationships was evaluated according to *r* and the significance value (*p*) level. For each date of acquisition, *r* values were calculated using the following equation:

$$r=\frac{\sum_{i=1}^{n}\left(x_{i}-\bar{x}\right)\left(y_{i}-\bar{y}\right)}{\sum_{i=1}^{n}{\left(x_{i}-\bar{x}\right)}^{2}\sum_{i=1}^{n}{\left(y_{i}-\bar{y}\right)}^{2}}$$

where $x_{i}$ and $y_{i}$ are the vegetation index and observed yield value for observation *i*, respectively; $\bar{y}$ and $\bar{x}$ represent the mean of the vegetation index and observed yield; and *n* is the number of observations.

The graphs related to the fluctuation of these *r* values among days after planting (DAPs), including significance levels of *p* < 0.05, were generated to assess the sensitivity of each vegetation index and satellite image to yield estimation throughout the growing season. The R^2^ and Root Mean Square Error (RMSE) parameters were used to evaluate the estimation.

$$R^{2}=1-\frac{\sum_{i=1}^{n}{\left(y_{i}-{\hat{y}}_{i}\right)}^{2}}{\sum_{i=1}^{n}{\left(y_{i}-{\bar{y}}_{i}\right)}^{2}}$$

and

$$RMSE=\sqrt{\frac{\sum_{i=1}^{n}{\left(y_{i}-{\hat{y}}_{i}\right)}^{2}}{n}}$$

where $y_{i}$ and ${\hat{y}}_{i}$ are the observed and predicted yield values for observation *i,* respectively; ${\bar{y}}_{i}$ is the mean observed value.

## 3. Results and Discussion

### 3.1. Time-Series Vegetation Index and Phenological Stage

**Figure 3:**
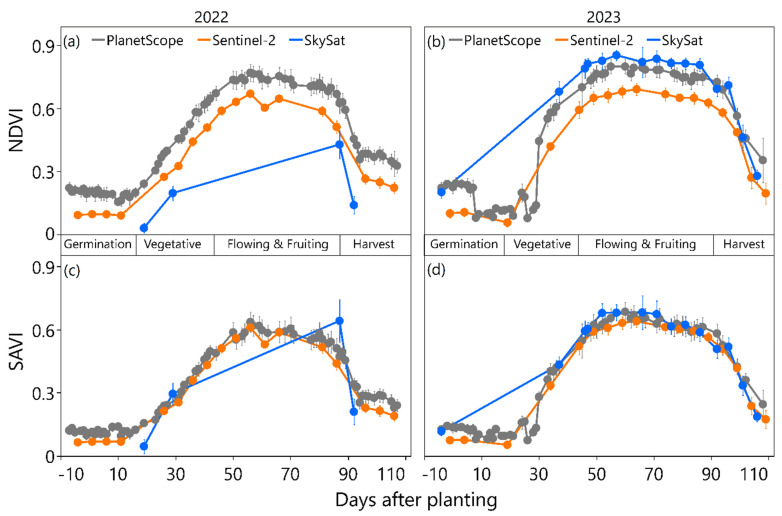
Japanese squash growth according to the two vegetation indices derived from the Sentinel-2 (orange line), PlanetScope (grey line), and SkySat (blue line) images from planting to harvest for the 2022 (**a**,**c**) and 2023 (**b**,**d**) growing seasons. The data points represent the average NDVI and SAVI values for the pixels that overlay the experimental strips. The error bars indicate the standard deviations of the indices. The average number of pixels for each satellite is 9, 904, and 30005 for Sentinel-2, PlanetScope, and SkySat, respectively. The different phenological stages of crop growth (germination, vegetative, flowering and fruiting, and harvest) are marked.

Figure 3 presents the time series of the vegetation indices derived from various remote sensors for the growing seasons of 2022 and 2023. In terms of growth curve characterization, the NDVI and the SAVI indicate similarities in the overall trends and stages of vegetation development. At the germination stages, both the NDVI and SAVI values are at their lowest. The NDVI and SAVI values gradually increased as the canopy grew denser and healthier as the squash plants produced leaves, stems, and vines about 20 days after planting. This marked the start of the vegetative stages and significant Japanese squash growth. After approximately 50 days of planting, the growth of Japanese squash reached its peak, which is associated with the highest vegetation indices values. During the flowering and fruiting stages (from around 50 to 80 days after planting), the NDVI and SAVI values slightly fluctuate, but generally remain high, which is indicative of healthy vegetation. Then, the NDVI and SAVI values slightly decrease due to the diversion of resources towards fruit production rather than vegetative growth. About 90 days after planting, the NDVI and SAVI values decline as the fruits mature and ripen, reflecting plant senescence and canopy deterioration. Finally, when Japanese squash reached full maturity and was harvested, the vegetation indices values dropped to their lowest point.

### 3.2. Correlation Between Yield and Vegetation Index

**Figure 4:**
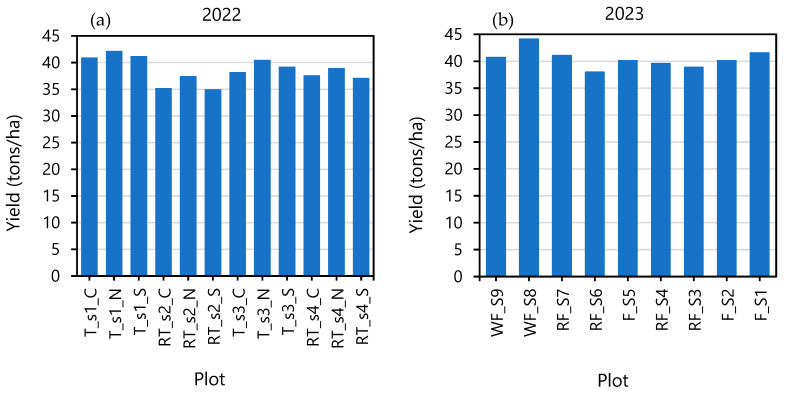
Within-field variations in squash yield for (**a**) 2022 and (**b**) 2023 experiments. T = full till, RT = reduced till, WF = winter fallow, s = strip line.

Figure 4 shows the within-field experimental plot variations in squash yield for the 2022 and 2023 growing seasons. The average yield for 2022 was 40.29 tons/ha, with the RT_s2_C plot recording the minimum yield (32.28 tons/ha) and the T_s1_N plot recording the maximum (46.57 tons/ha). The average yield for the 2023 experiment was 40.29 tons/ha, with the RF_S6 plot recording the minimum yield (37.97 tons/ha) and WF_S8 recording the maximum (44.13 tons/ha). The full tillage treatments exhibited higher yields, likely due to improved soil aeration, water infiltration, and nutrient availability. In contrast, the reduced tillage treatments showed lower yields, potentially due to restricted root growth and nutrient uptake. These findings align with the previous studies emphasizing the importance of soil management in optimizing crop productivity [1,21].

**Figure 5:**
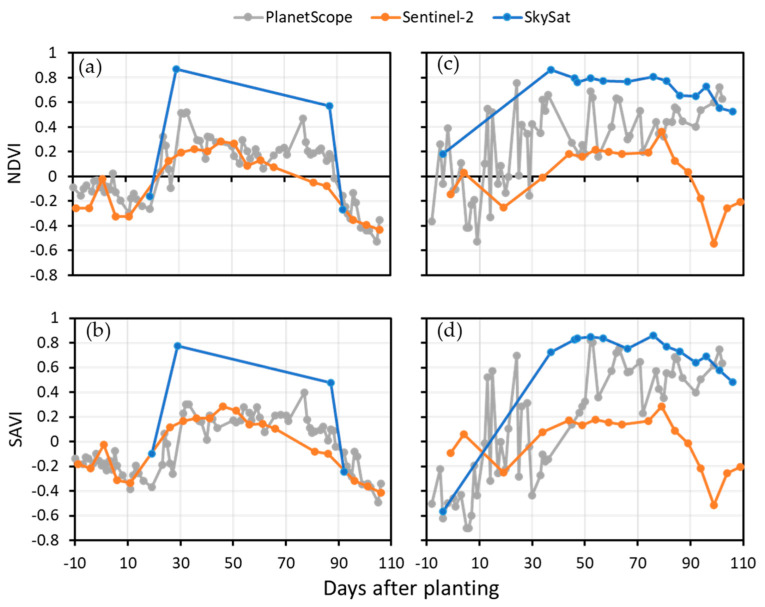
Correlation of Japanese squash yield and vegetation index of NDVI (top) and SAVI (bottom) by date for growing seasons of 2022 (**a**,**b**) and 2023 (**c**,**d**), respectively.

Figure 5 illustrates the temporal fluctuations in correlation between the ground-based yield measures and the vegetation indices extracted from the different remote sensors. The SkySat-derived vegetation indices exhibited the highest correlation coefficient, given the correlation values ranged from −0.27 to 0.87 and 0.19 to 0.86 for the 2022 and 2023 growing seasons, respectively. This can be attributed to the high spatial resolution of SkySat images (i.e., 0.5 m) that enabled the reduction in the generalization in crop spectral reflection and the delineation of strip boundaries, so that noise from the neighboring field was removed.

The correlations between the yield and SkySat-derived vegetation indices for both the 2022 (Figure 5a,b) and 2023 (Figure 5c,d) growing seasons changed accordingly with the temporal variation in the NDVI and the SAVI, as shown in Figure 3. In 2022, the yield was best explained (i.e., it showed the highest value of *r*) by the NDVI and SAVI measurements taken 29 days after planting (*r* = 0.87 and 0.77, respectively). Fairly significant correlations were still found with the NDVI measurements taken 87 days after planting (*r* = 0.57). In 2023, the correlation between the SkySat-derived NDVI and SAVI and squash yield becomes notably significant from the middle of the vegetative stage and extends throughout the flowering and fruiting stages. This marks the period of highest correlation values with yield, which generally coincides with the peak of the vegetation indices curve. The most robust correlations between the ground-based yields and the NDVI (*r* = 0.86) were observed 37 days after planting. However, the strongest correlation between the ground-based yields and the SAVI (*r* = 0.85) was observed 76 days after planting, which is generally the same time when the growth of Japanese squash reached its peak. These findings indicate that the timing of the maximum correlation between the yield and vegetation indices can vary between fields and growing seasons due to the variations in agricultural management and environmental conditions (e.g., temperature).

The vegetation indices extracted from the PlanetScope also exhibited strong correlations with yield in the 2023 growing season. The most robust correlations between the ground-based yields and the NDVI (*r* = 0.76) were observed 24 days after planting, which is generally the same time as the initiation of the vegetative stages (as shown in Figure 3b). However, the strongest correlation between the ground-based yields and the SAVI (*r* = 0.81) was observed 52 days after planting, which is generally the same time when the growth of Japanese squash reached its peak. A notable difference between the NDVI- and SAVI-derived models was that the relationship between yield and the NDVI was significant during the beginning of the heading stage (20–30 days after planting), while the SAVI exhibited higher coefficient values during the flowering and fruiting stages (approximately 60–80 days after planting). The differential performance of the NDVI and the SAVI in detecting yield can be attributed to their sensitivity to the soil background and canopy density. In sparsely vegetated ground, especially in early growth stages, the NDVI failed to account for soil reflectance and overestimated vegetation health. In contrast, the SAVI incorporates a soil adjustment factor to provide more accurate estimations during these stages by minimizing the soil background effects. This distinction is important for precision agriculture, as the SAVI is a more reliable index for early yield estimation in fields with partial canopy cover [11].

The Sentinel-2-derived vegetation indices exhibited a weaker correlation with squash yield regardless of the time for both the 2022 and 2023 growing seasons. Despite providing richer spectral information in the visible and near-infrared spectra (8 bands) compared to PlanetScope (4 bands), Sentinel-2 did not identify the optimal timing for correlating yield with the surface reflectance data. This indicates that in-season squash yield prediction using Sentinel-2, particularly for small farms, may not be reliable. The lower accuracy of the vegetation indices derived from Sentinel-2 can be attributed to its moderate spatial resolution (10 m), which limits its ability to capture fine-scale field variability. The broader wavelength range of Sentinel-2’s NIR band (770–910 nm) compared to SkySat’s NIR band (780–860 nm) may also contribute to the differences in the vegetation index calculations. However, the primary factor affecting Sentinel-2’s performance is its inability to resolve fine-scale field variability, particularly in small plots (<2 ha). This is consistent with the previous studies that demonstrated that satellite imagery with a 30 m resolution (e.g., Sentinel-2 and Landsat-8) often fails to resolve the field-scale yield variability that is important for precision agriculture applications, especially for smaller fields [13,22].

Another noticeable, albeit weak, feature was the negative correlation of the NDVI and the SAVI to yields at germination and the beginning of the vegetative period. Bare soil reflectance has been reported to correlate with soil properties such as soil moisture, soil organic carbon, and clay content [23,24]. At the field scale, these relationships are often site-specific. [23] reported a negative correlation of the soil NDVI with the topsoil clay content and a positive correlation with the topsoil organic carbon content. Conversely, [24] reported a negative correlation between the NDVI and the soil organic carbon. Consequently, when a strong relationship between these properties and yield is observed, one may expect the bare soil NDVI and yield to be site-specific, as [23] found a positive correlation between the bare soil NDVI and maize yield in salt-affected farmland in coastal northern Italy.

### 3.3. Predicting Yield

The results of the statistical analysis presented in Figure 5, particularly concerning small-scale plots akin to those utilized in our study, reveal the notable impact of spatial resolution on the accuracy of squash yield estimation via the NDVI and the SAVI in the small-scale plot trials. In particular, the correlation coefficients (r values) related to yield estimation are strongly influenced by the resolution of satellite pictures from SkySat (0.5 m), PlanetScope (3 m), and Sentinel-2 (10 m). Compatibility between the spatial resolution of the satellite images and the size of the experimental plots emerges as a crucial determinant for accurate yield estimation. When it comes to yield predictions, SkySat shows remarkable dependability in small-scale fields. This reliability facilitates precision agriculture applications and aids in early-season through pre-harvest decision-making by providing insights into expected yield distribution across a field.

**Figure 6:**
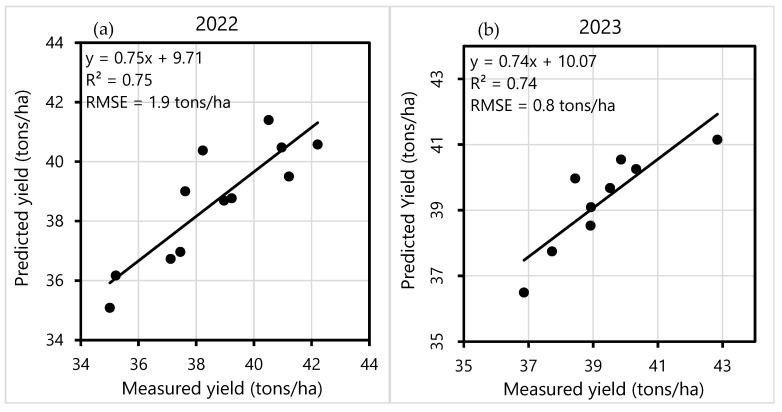
The comparison between the actual yields and the estimated yields for the growing seasons of (**a**) 2022 and (**b**) 2023 using linear regression with the NDVI data derived from the SkySat images collected on the 29th and 37th days after planting, respectively.

The scatter plot in Figure 6 displays the relationship between the vegetation indices and squash yield at the peak correlation period. These plots illustrate the regression relationships established between the observed yields across the different strips and the corresponding yields predicted based on the NDVI derived from the SkySat images. For the 2022 growing season, the correlation between predicted and measured yield yielded a favorable R^2^ value of 0.75. However, it is important to note that the model’s accuracy diminished for lower yields. The model for the 2023 growing season exhibited precision comparable to that of 2022, with an R^2^ value of 0.74. For the 2022 and 2023 growing seasons, the RMSE values of the early estimate were 1.9 and 0.8 tons/ha, respectively. The low RMSE value of yield prediction suggests that these early-season predictions are reliable. In both years, the constancy of the relationships between the squash yield and vegetation indices during the same growth stage (i.e., the start of the heading stage) indicates the predictive capacity of SkySat. Nevertheless, our results suggested that the relationships from one year could not be used to predict yield the following year. Further research is needed to determine if in-season vegetation indices can serve as qualitative yield indicators, or if more precise forecasting models can be developed. The absence of cross-validation and out-of-one-year validation limits our findings’ generalizability, highlighting the necessity of multi-year datasets and a range of environmental variables to evaluate the methodology’s stability and consistency. Future studies should incorporate these elements to strengthen the robustness and applicability of vegetation index-based yield predictions across varying temporal and spatial scales.

For small-scale farms, the early detection of within-field variability is essential science it enables timely interventions that can significantly reduce potential yield losses. These patterns are demonstrated by the supplemental spatial maps of yield prediction (Supplementary Materials), which illustrate within-field variability. Smallholder farmers can prevent small problems from becoming more serious by optimizing management measures, such as insect control, fertilization, and irrigation. This targeted approach is particularly important for small farms, where even minor yield reductions can have substantial financial consequences [25,26]. Furthermore, optimizing resource use through precise interventions reduces costs and improves sustainability by conserving essential resources, minimizing the environmental impact, and supporting long-term soil health [27]. The earlier yield forecasting information is provided, the more effectively farmers can apply proactive steps to mitigate yield losses and ensure a successful harvest [28].

The selection of a remote sensing platform significantly influences the accuracy of yield prediction models in small farms. The satellite imagery from Sentinel-2, PlanetScope, and SkySat, each offering different spatial resolutions, is central to this process. Sentinel-2, with a 10 m resolution, provides a broader perspective that is beneficial for large-scale monitoring and analysis. Although the open-access nature of the Sentinel-2 data presents a significant advantage, its lower resolution may limit its effectiveness in capturing fine-scale variability within small plots [29]. With daily coverage and 3-m resolution, PlanetScope imagery offers a unique combination of a high spatial resolution and frequent temporal updates. This makes it especially valuable for monitoring changes over time. However, the lack of atmospherically corrected or radiometrically calibrated radiance data may limit the ability to reliably characterize the changing conditions of land surface features, and vegetation canopies specifically [30]. 

With its high resolution 0.5-m pixel, SkySat is ideal for small-scale farms since it can accurately estimate yields by catching intricate within-field spatial variations. This high level of detail supports accurate early-season and pre-harvest assessments, enhancing decision-making and intervention strategies in precision agriculture. Nonetheless, the high cost of SkySat may limit its accessibility for certain users and can be a considerable barrier, particularly for smaller operations or research projects with limited budgets. Another limitation of SkySat satellites, which are built on the CubeSat concept, is the frequent inconsistency in the data collected by different satellites within the constellation. This inconsistency may reduce the accuracy of surface reflectance-based applications, such as estimating vegetation indices, and could hinder the effectiveness of SkySat satellites in monitoring the changes on Earth’s surface [31].

The emergence of drones equipped with high-resolution sensors and cameras provides an additional tool for yield prediction. With spatial resolutions often surpassing those of satellite imagery, sometimes down to a few centimeters, drones offer remarkable detail, allowing for the capturing of fine-scale variations within small plots [32]. This high level of detail is advantageous for precise yield estimation and in-season crop growth monitoring. Additionally, drones also make it possible for regular and targeted data collection, which is essential for detecting changes and implementing timely interventions. Drone-based remote sensing is fast, convenient, and efficient for many applications because of its capacity to operate on-demand and in varied weather conditions. Affordable drone platforms coupled with user-friendly software are increasingly accessible to growers and consultants, potentially encouraging stakeholders’ adoption of precision agriculture technologies [33]. Unfortunately, while the technical specifications of drones are often ideal for precision agricultural applications, the cost of image acquisition and processing for large-scale operations is typically prohibitive, limiting their widespread adoption. Additionally, their limited coverage area per flight and potential regulatory constraints further restrict their broad-scale use [34]. Despite these limitations, drones remain a valuable tool for small-scale farms and targeted monitoring, complementing satellite-based approaches and offering a versatile solution for precision agriculture.

This study demonstrated the effectiveness of high-resolution remote sensing in yield prediction. This research did not examine the role of environmental variables, such as soil moisture and temperature intra- and inter-annual variability. Soil moisture affects crop water availability, influencing plant health and biomass accumulation, while temperature variations regulate growth stages and overall productivity. These factors can introduce variability in remote sensing-based yield predictions, particularly under fluctuating climatic conditions. Recent studies suggest that integrating soil moisture data from microwave remote sensing (e.g., SMAP and Sentinel-1) with optical indices like the NDVI and the SAVI could enhance model accuracy by accounting for water stress effects [35,36]. Likewise, incorporating temperature datasets from thermal infrared sensors (e.g., ECOSTRESS and MODIS) may help refine phenological stage assessments and stress detection [37]. Future research should explore combining optical, thermal, and microwave remote sensing with ground-based sensor networks to improve their explanatory power. Machine learning techniques could be leveraged to fuse these diverse datasets, offering a more comprehensive approach to yield estimation.

## 4. Conclusions

This study aimed to investigate the utility of three temporally and spatially detailed remotely sensed datasets for predicting Japanese squash yields during the growing seasons of 2022 and 2023 in Hollister, California. The datasets evaluated included the NDVI and the SAVI obtained from the Sentinel-2, PlanetScope, and SkySat sensors. The results indicated that the SkySat-derived vegetation indices outperformed the others in explaining squash yields. Conversely, the vegetation indices derived from Sentinel-2 exhibited the weakest correlations with squash yield due to its limited ability to capture within-field yields with a relatively low spatial resolution (i.e., 10 m), particularly for the small fields. The NDVI and the SAVI were strongly and positively correlated with squash yields, with yield estimation accuracy steadily increasing at the initiation of the vegetative stages. Another noticeable, albeit weak, feature was the negative relationship of the NDVI and the SAVI to squash yields at germination and the beginning of the vegetative stage. The satellite images availability varied annually and over time during the growing season. Considering the availability of cloud-free remotely sensed imagery, the accuracy of crop yield prediction fluctuates throughout the growing phase. Therefore, determining the stage of crop growth at which satellite imagery provides the most accurate estimation is crucial for enhancing yield prediction accuracy. In our study, the most accurate yield estimation was generated by the vegetation indices derived from the SkySat images collected on the 29th day after planting in 2022, while in 2023, the 37th and 76th days after planting resulted in optimal results for the NDVI-based and SAVI-based estimations, respectively.

In summary, this study underscores the benefits of utilizing high-resolution satellite imagery to estimate within-field yield. Ensuring compatibility between the spatial resolution of images and the scale of experimental plots is crucial for accurate yield estimation in agricultural settings. Enhanced spatial resolution enables the more precise monitoring of agricultural practices both within and between plots. SkySat proves to be highly valuable for precision agriculture applications, adept at identifying the variations in yield within a field and having the potential to inform strategic management decisions geared towards maximizing profitability. However, in our study, the scale of yield variability was limited due to the imposed experimental treatments. We expect that in a homogeneously managed fields, even a relatively small one, SkySat may perform better than other systems.

Additionally, the level of estimation accuracy will change according to the time of satellite image acquisition, and this study demonstrated the importance of capturing imagery during the critical stages of crop development, such as the initiation of vegetative growth, when crop canopies are denser and healthier, which is essential for early yield forecasting. Further analysis and related studies are required to determine precisely how much information gain is achieved by regular, as opposed to occasional, in-time model updating. More importantly, the broader utility of high-resolution satellite images to aid in the field management and decision-making process should be further discussed.

## Figures and Tables

**Table 1:** Satellite images collected during the Japanese-squash-growing season in 2022 and 2023.

Satellite	2022	2023
Sentinel-2	May: 14, 19, 24, 29 June: 13, 18, 23, 28 July: 3, 8, 13, 18, 23, 28 August: 2, 7, 12, 22, 27	May: 19 June: 3, 18, 28 July: 3, 8, 13, 18, 28 August: 2, 7, 12, 17, 22, 27
PlanetScope	May: 15–24, 26, 28–31 June: 1, 3, 6, 10–14, 18–20, 22, 24, 25, 27–29 July: 1, 4, 7, 8, 10, 11, 13, 14, 16, 17, 19, 23, 25, 27, 28 August: 3–7, 9, 10, 12–15, 18–23, 25, 27, 28	May: 15, 16, 18, 20–24, 27–30 June: 1, 2, 4, 5, 8–10, 12–14, 17–20, 22, 26, 27, 29 July: 2–4, 6, 7, 9, 14, 16, 17, 20, 21, 25, 26, 31 August: 1, 3, 4, 6–8, 10, 15, 17, 22, 24, 25
SkySat	June: 6, 16 August: 13, 18	May: 11 June: 21, 30 July: 1, 6, 11, 20, 25, 30 August: 4, 9, 15, 19, 24, 29

**Table 2:** Vegetation indices calculated using satellite imagery.

Vegetation Indices	Equation	References
Normalized Difference Vegetation Index (NDVI)	$\frac{NIR-Red}{NIR+Red}$	[20]
Soil-Adjusted Vegetation Index (SAVI)	$\frac{NIR-Red}{NIR+Red+L}*\left(1+L\right)$	[11]

L is soil brightness correction factor, which was set at 0.5 because of intermediate vegetation cover [11].

## Data Availability

The raw data supporting the conclusions of this article will be made available by the authors, without undue reservation.

## Supplementary Materials

The following supporting information can be downloaded at: https://www.mdpi.com/article/10.3390/s25071999/s1.
