# Supplementary material for: In-Season Estimation of Japanese Squash Using High-Spatial-Resolution Time-Series Satellite Imagery
Source: Sensors (Basel). 2025 Mar 22;25(7):1999. doi: 10.3390/s25071999 (PMC11991110; doi:10.3390/s25071999)
Supplement: Supplementary file 1 [file sensors-25-01999-s001.zip › sensors-3457257-supplementary.pdf]

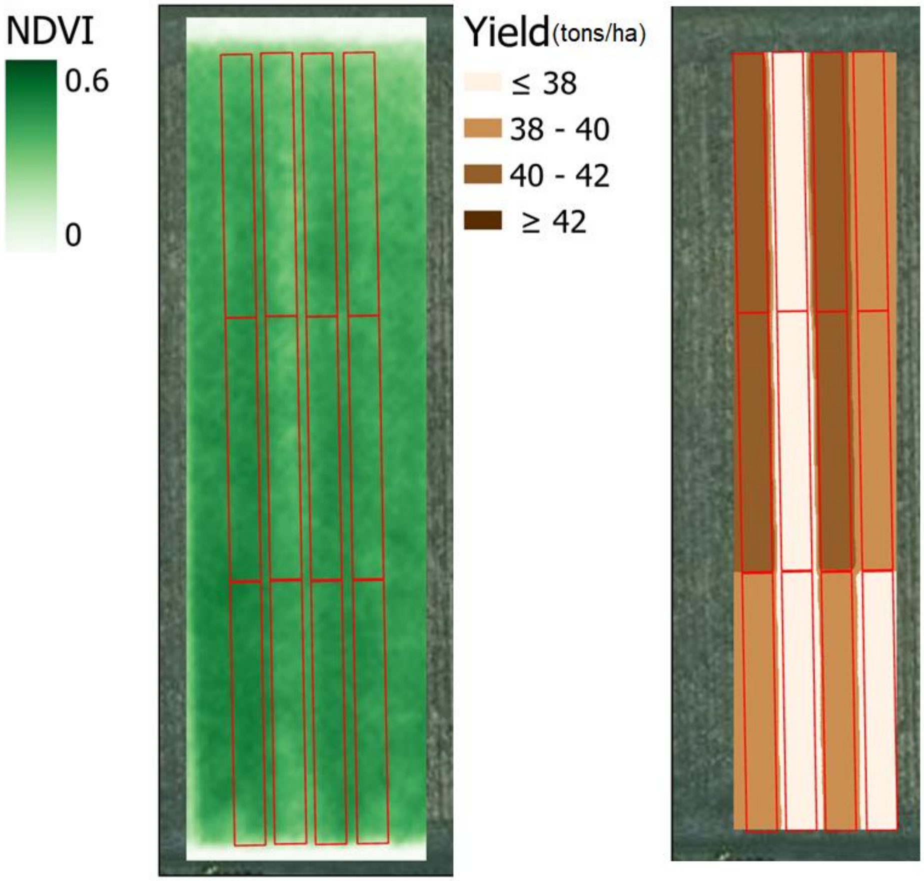

Supplementary Figure S1 Predicted yield map generated for 2022 based on the NDVI derived from SkySat images collected on the 29th day after planting.

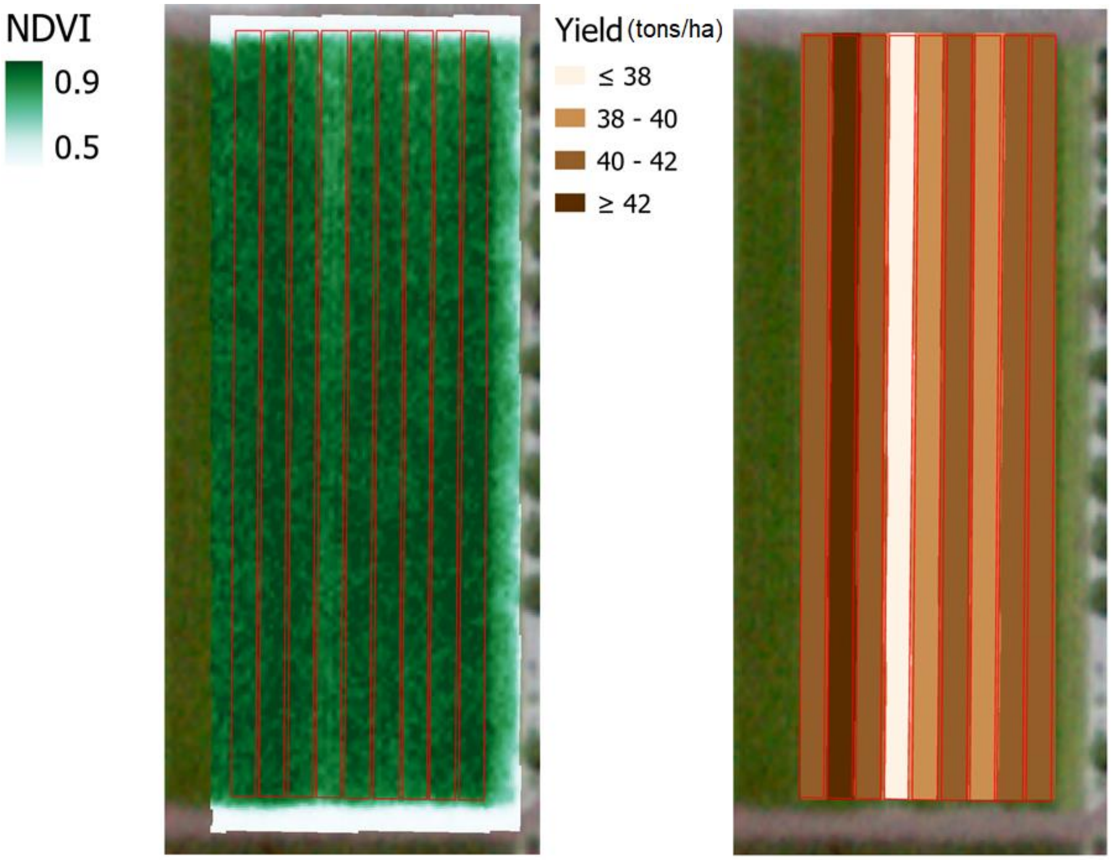

Supplementary Figure S2 Predicted yield map generated for 2023 based on the NDVI derived from SkySat images collected on the 37th day after planting.
